# Comparison of Magnetic and Transcutaenous Tibial Nerve Stimulation Added to Bladder Training for Overactive Bladder: A Randomized Controlled Trial

**DOI:** 10.1007/s00192-025-06215-w

**Published:** 2025-07-07

**Authors:** Necmettin Yildiz, Saadet Nur Sena Oztekin, Yesim Akkoc

**Affiliations:** 1 Faculty of Medicine, Department of Physical Medicine and Rehabilitation, Pamukkale University, Denizli, Turkey; 2 Faculty of Medicine, Department of Physical Medicine and Rehabilitation, Ege University, İzmir, Turkey

**Keywords:** Idiopathic overactive bladder, Bladder training, Magnetic stimulation, Transcutaneous tibial nerve stimulation

## Abstract

**Introduction and Hypothesis:**

This study aimed to compare the efficacy of magnetic stimulation (MStim) and transcutaneous tibial nerve stimulation (TTNS), both combined with bladder training (BT), in women with idiopathic overactive bladder (iOAB).

**Methods:**

Women with iOAB were randomized into three groups: group 1 received BT only; group 2 received MStim plus BT; and group 3 received TTNS plus BT. MStim and TTNS were administered twice weekly, 30 min per session, for a total of 12 sessions over 6 weeks. The primary outcome was the positive response rate, defined as ≥ 50% fewer incontinence episodes, measured via a 3-day bladder diary at 6 weeks. Seconder outcomes included incontinence severity (pad test), voiding frequency, incontinence episodes, nocturia, number of pads used, symptom severity (OAB-V8), QoL (IIQ-7).

**Results:**

According to the sample size calculation, 66 women were included in the study, with 22 assigned to each group. The three groups were comparable in terms of demographic and clinical characteristics. The positive response rate (primary outcome) was significantly higher in groups 2 and 3 compared to group 1 at week 6 (66.7% and 90.5% vs. 35.0%, *p* = 0.001), with no significant difference between groups 2 and 3 (*p* = 0.130). Both stimulation groups (each *n* = 21) demonstrated significant improvements in incontinence severity, incontinence episodes, number of pads used, symptom severity, QoL, and treatment satisfaction compared to the BT-only group (*n* = 20) (*p* < 0.0167). No significant differences were found between group 2 and group 3 for these parameters. Voiding frequency significantly improved in group 3 (med 11 to 6) compared to group 1 (med 11 to 8) and group 2 (med 12 to 8) (*p* < 0.0167).

**Conclusions:**

Both MStim plus BT and TTNS plus BT are more effective than BT alone in women with iOAB. These two stimulation methods have similar clinical efficacy, with TTNS demonstrating greater effectiveness in reducing voiding frequency.

**Clinical trial registration:**

This study was registered with ClinicalTrials.gov number, NCT05387824.

## Introductıon

Idiopathic overactive bladder (iOAB) is a common condition in women, characterized by urinary urgency, frequency, nocturia, and urgency urinary incontinence, without identifiable neurological or structural pathology [1]. First-line management of iOAB includes conservative therapies such as bladder training (BT) and pelvic floor muscle (PFM) exercises. When these are insufficient, second-line treatments such as pharmacotherapy (e.g., antimuscarinics, beta-3 agonists) and neuromodulation techniques are considered [2–4].

Among neuromodulation approaches, transcutaneous tibial nerve stimulation (TTNS) and magnetic stimulation (MStim) have gained attention due to their noninvasive nature and ease of application. TTNS has shown efficacy in alleviating iOAB symptoms and improving quality of life (QoL), with advantages such as reduced preparation time and patient comfort compared to percutaneous methods [7–10]. Similarly, MStim has been associated with improvements in incontinence symptoms and QoL, although the evidence is still evolving and marked by heterogeneity across studies [13–18]. However, there is a lack of clear data on their comparative efficacy in the treatment of iOAB. To our knowledge, there is a lack of randomized controlled trials directly comparing MStim and TTNS in women with iOAB.

This study aims to evaluate and compare the effects of TTNS and MStim, when combined with BT, on urinary incontinence symptoms and QoL in women with iOAB.

## Material Methods

### Study Design

This prospective, randomized controlled trial was conducted at the Urogynecological Rehabilitation Unit of Pamukkale University Hospital, Department of Physical Medicine and Rehabilitation, between July 2021 and July 2023. Ethical approval was obtained from the Institutional Review Board of Pamukkale University (approval number: 60116787–020/37878), and the study was registered at ClinicalTrials.gov (NCT05387824). The study protocol adhered to the CONSORT 2010 guidelines.

### Population and Sample

Women presenting with symptoms suggestive of iOAB were referred to the Urogynecological Rehabilitation Unit from associated outpatient clinics. Eligible participants were identified through structured face-to-face clinical interviews by a physician (OSNS). The diagnosis of iOAB was based on clinical history, absence of neurological or anatomical causes, normal urinalysis, and the presence of urgency, frequency, and/or urgency incontinence symptoms in the absence of urinary tract infections or structural abnormalities. All participants were informed about the study’s purpose and procedures and provided written informed consent.

Inclusion criteria were: women over 18 years old with a clinical diagnosis of iOAB who did not tolerate or had no response to antimuscarinic medications (discontinued for at least 4 weeks), were able to comprehend the study procedures and complete the voiding diary and QoL questionnaires, and had a PFM strength of ≥ 3/5. PFM strength was assessed via digital vaginal palpation by a single trained physician (OSNS), in accordance with the modified Oxford Grading Scale [21].

Exclusion criteria included: stress urinary incontinence (clinically evaluated), previous treatments with BT, MStim or TTNS/PTNS, recent urogynecological surgery (last 3 months), pregnancy or intention to become pregnant, vulvovaginitis or urinary tract infection, malignancy, POP-Q stage > 2 [22], anatomical malformations or skin lesions precluding electrode application, implanted cardiac or neurological devices, ongoing arrhythmia treatment, undiagnosed abdominal pain or dysmenorrhea, metal implants or devices between lumbar and lower extremities, and neurological disorders. Women with post-void residual volume > 100 ml on portable bladder ultrasonography (Telemed Micrus, Lithuania) were also excluded.

### Randomization and Blinding

Participants were randomly assigned in a 1:1:1 ratio using a computer-generated randomization list to one of three groups: group 1 (BT alone), group 2 (BT + MStim), and group 3 (BT + TTNS). Allocation was performed by an independent researcher not involved in outcome assessments. Outcome evaluations were performed by a physician (YN) blinded to the group assignments. The interventions were administered by a single experienced physician (OSNS).

### Interventions

All participants first received a standardized 30-min BT education session and a written home-based protocol. The standardized BT program, consisting of four stages detailed in previous studies [4, 5, 18, 23, 24], was implemented for 6 weeks by an experienced urogynecological rehabilitation nurse (Table 1). The interventions performed in each group are described below.

**Table 1:** Bladder training (BT) protocol administered to patients

Stage	Component	Details of intervention
1	Initial education and PFM awareness	- At the initial visit, patients received information about basic pelvic anatomy and bladder function - Patients were taught how to locate their pelvic floor muscles (PFM) - Each patient was shown how to contract the PFM at least once through digital vaginal palpation - This was only for awareness; no structured PFM exercise program was provided
2	Urgency suppression techniques	- Patients were trained to manage urgency using the following techniques: • Stop the activity immediately and sit down if possible • Relax the whole body • Perform several quick repeated PFM squeezes • Take deep breaths • Distract the mind with another task • Use positive self-talk (e.g., “I can hold it,” “I am in control”)
3	Timed voiding program	- A scheduled voiding routine was introduced in two steps: 1. Start urinating at fixed time intervals based on a bladder diary (e.g., every 2 h) 2. Gradually increase the interval between voids by 15–30 min every few days, aiming to expand bladder capacity and improve control
4	Motivation and continuation support	- Patients were encouraged to continue using BT as a long-term strategy - They were informed that this is an effective behavioral treatment for urinary urgency - Motivation and adherence were supported through regular follow-up and reinforcement of progress

The control group (group 1, BT-only) received the standard BT protocol with no additional interventions.

**Fig. 1:**
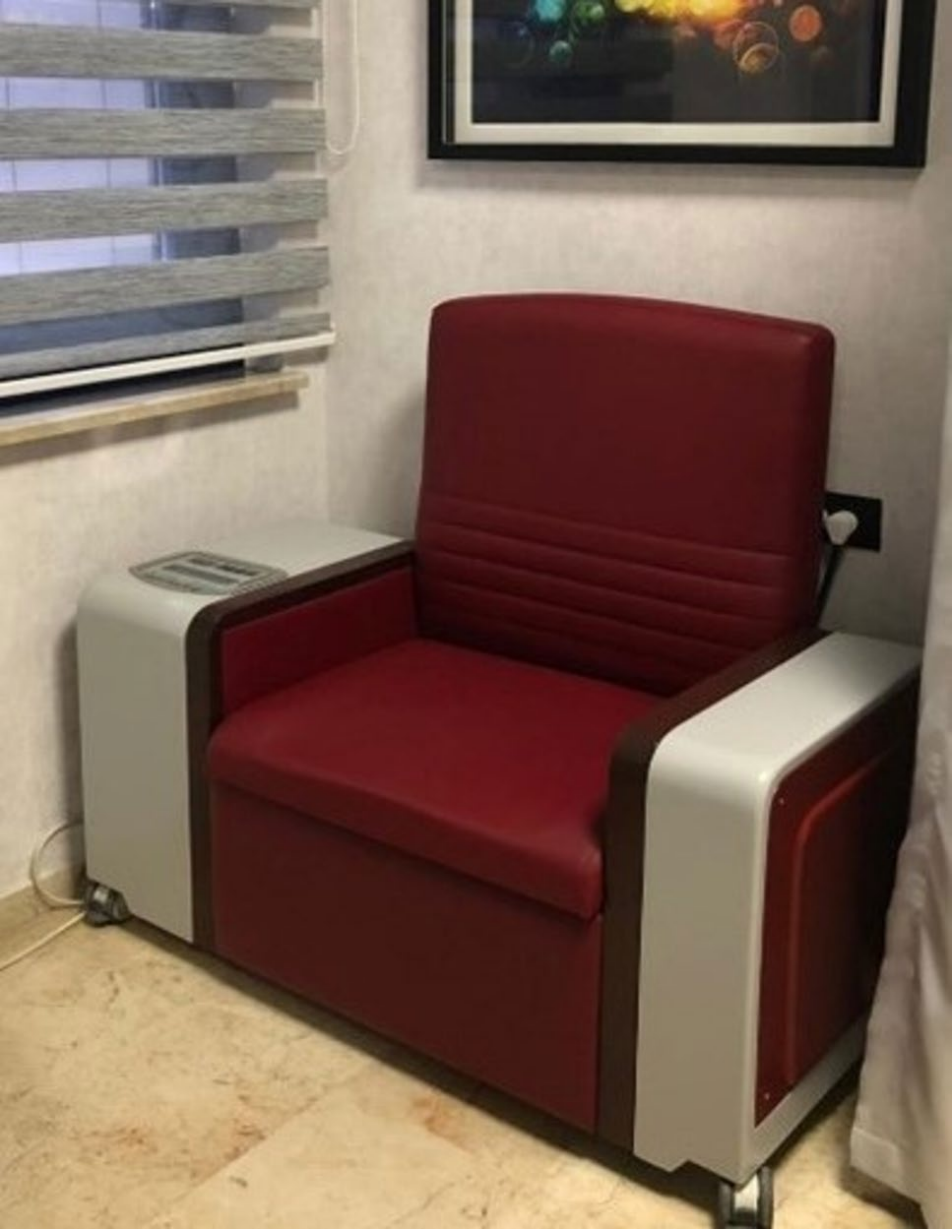
The chair-type magnetic stimulator (Novamag NT60)

The BT plus MStim group (group 2) received combined BT and MStim using an armchair-type stimulator (Novamag NT60, Turkey) (Fig. 1). Participants were informed about the MStim mechanism, treatment goals, and their role during sessions. They were instructed to sit on the armchair with a magnetic coil positioned beneath it. This generated an eddy current flow, stimulating the nerves or muscles of the pelvic floor. The device was set to deliver maximum stimuli, with a pulse width of 200 μs and a repetition cycle of 10 Hz, in accordance with previous research [13, 14, 18]. At each session, the device was adjusted to ensure participants received the maximum tolerable stimulation intensity [6, 13, 14, 17, 18]. MStim was administered twice weekly, 30 min per session, for a total of 12 sessions over 6 weeks.

**Fig. 2:**
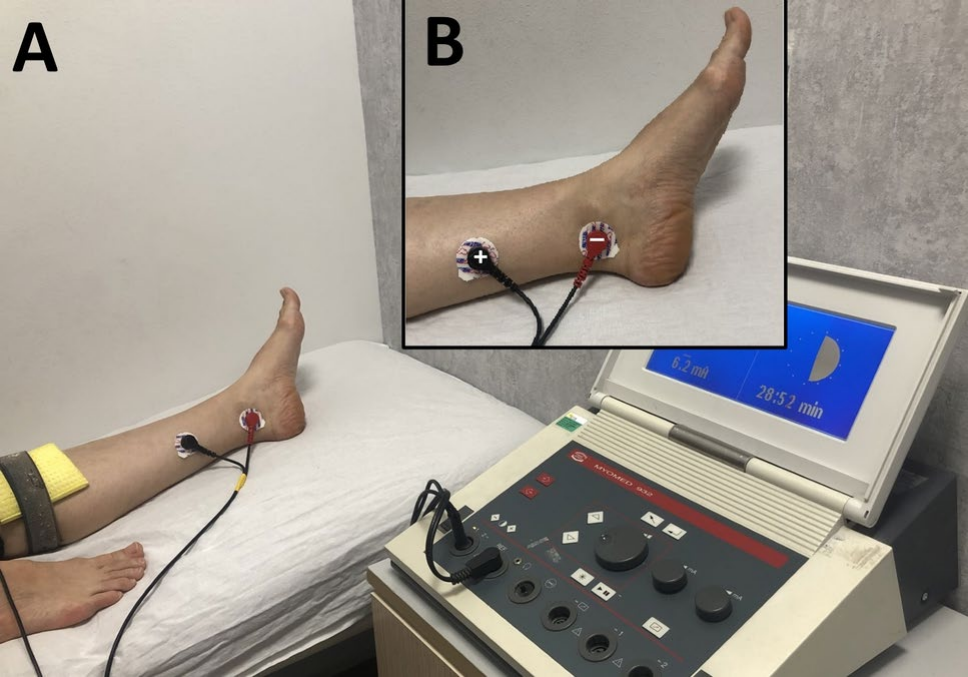
(**A**) Patient and electrical stimulation equipment positioning for transcutaneous tibial nerve stimulation (TTNS) (**B**) Electrodes positioning for TTNS

The BT plus TTNS group (group 3) received BT combined with TTNS. The stimulation was performed unilaterally (left limb as the standard), in the supine lying position using a stimulation device (Enraf Nonius Myomed 932) with surface electrodes. Two round self-adhesive surface electrodes with a diameter of 33 mm were positioned according to established protocols, with the negative electrode 2 cm behind the medial malleolus and positive electrode 10 cm proximal to it [9, 10]. Ground electrode was placed on the ipsilateral extremity (Fig. 2). The stimulation protocol was delivered at fixed of 20 Hz and pulse width of 200 ms in continous mode in accordance with tibial nerve stimulation protocol [7, 8]. Correct positioning was determined by gradually increasing the stimulation current intensity (range 0–50 mA) from 2 mA in 1 mA increments and noting a hallux reaction (plantar flexion of the great toe or fanning of all toes), after which the final stimulation intensity was set based on the subject’s comfort level. TTNS sessions were performed two times in a week, for 6 weeks. Every session lasted 30 min. The intervention comprised a 12 session treatment program of TTNS [7, 8].

All treatment sessions (MStim and TTNS) were performed and monitored by the same physician (OSNS). Participants’ adherence to BT was monitored using biweekly bladder diaries and daily checklists reviewed by the urogynecological rehabilitation nurse.

### Outcome Measures

The primary outcome was the positive response rate, defined as a ≥ 50% reduction in incontinence episodes (times/day), assessed using a 3-day bladder diary [18, 24]. Secondary outcomes included incontinence severity, voiding frequency, nocturia, number of pads used, symptom severity, and QoL. All participants were interviewed by the physician administering TTNS and MStim regarding any potential side effects associated with these treatments. Although there are no serious adverse events associated with transcutaneous stimulations, possible adverse events may include skin allergic reaction, redness, and irritation at the electrode application site, and stimulation related sensory complaints (pain, burning, tingling or itching) at the stimulation area. Additionally, participants were monitored for potential side effects related to MStim, including numbness in the lower extremities, muscle weakness, pain in the PFM and surrounding areas, changes in defecation-associated discomfort, headache, somnolence, malaise, and asthenia.

A 24-h pad test was used to evaluate incontinence severity [27]. Typically, the 24-h pad weight test was conducted without standardized activity instructions, as recommended by the ICS. However, patients were instructed to perform their usual physical activities. They began the test with an empty bladder and wore a pre-weighed standard waterproof pad inside their underwear. They were advised to change the pad every 4 to 6 h. The used pads were either weighed immediately or stored in an airtight bag for later weighing in a laboratory [27]. A 3-day bladder diary was used to collect data on voiding frequency (times/day), nocturia (times/night) and pad usage (times/day). The Overactive Bladder Questionnaire (OAB-V8) was used to assess OAB symptom severity [28, 29]. This self-administered 8-item questionnaire employs a Likert scale ranging from 0 (none) to 4 (too many), with a total score ranging from 0 to 40. Additionally, the self-administered Incontinence Impact Questionnaire (IIQ-7) was used to evaluate incontinence-specific quality of life (QoL) [30]. Higher scores on the IIQ-7 indicate worse QoL. Participants rated their satisfaction with treatment on a 5-point Likert scale (5 = very satisfied, 1 = very unsatisfied) [24].

Adverse events related to MStim and TTNS were queried and documented during each session. All assessments at baseline and at the end of treatment (week 6) were conducted by a different physician (YN), who was blinded to the group allocation, with the exception of response rates and treatment satisfaction, which were evaluated only at week 6.

### Compliance and Follow-Up

To maintain the timed voiding component of the BT program, participants in all groups completed a 3-day bladder diary every 2 weeks. Compliance with BT was monitored through daily checklists and biweekly bladder diaries. These diaries were reviewed every 2 weeks by our urogynecological rehabilitation nurse, ensuring the continuity of this program on an individual basis. Participants missing any therapy sessions (group 2 and group 3) or completing < 80% of BT checklists were excluded from the final analysis. All other medications unrelated to iOAB were continued unchanged. Participants were not instructed to modify their fluid intake habits, and no interventions were implemented to alter their habitual fluid consumption patterns during the study period. Consequently, fluid intake was neither accounted for nor normalized in the analysis.

### Statistical Analysis

A sample size calculation was performed on the basis of integer mean values reported in a previous study, where tibial nerve stimulation reduced incontinence episodes from 8 to 2 in the TTNS group and from 6 to 5 in the control group [9]. With a significance level of 95% (α = 0.05), power of 85% (β = 0.15), and an expected improvement of ≥ 50% in incontinence episodes over 24 h in the treatment groups compared to the control group, the optimal sample size was determined to be 20 women per arm. Accounting for a 10% withdrawal rate, 66 women (22 per group) were enrolled. Sample size calculation was conducted using G*Power 3.1 Statistical Power Analysis software.

The statistical analysis was conducted by another physician who was blinded to the group allocation (AY). SPSS 17.0 (SPSS, Chicago, IL) was used for statistical analysis. In each group, measurable parameters were tested with the Kolmogorov–Smirnov test evaluating normal distribution. Consequently, as none of the parameters exhibited a normal distribution, nonparametric tests were employed for all statistical analyses. Kruskal–Wallis variance analysis and ANOVA with the post-hoc Bonferroni correction (Mann–Whitney-Utest) and chi-square test were used for inter-group comparisons. Wilcoxon tests were used for intra-group comparisons. Statistical significance was set at *p* < 0.0167 for Bonferroni correction Mann–Whitney-Utest and *p* < 0.05 for other tests.

## Results

**Fig. 3:**
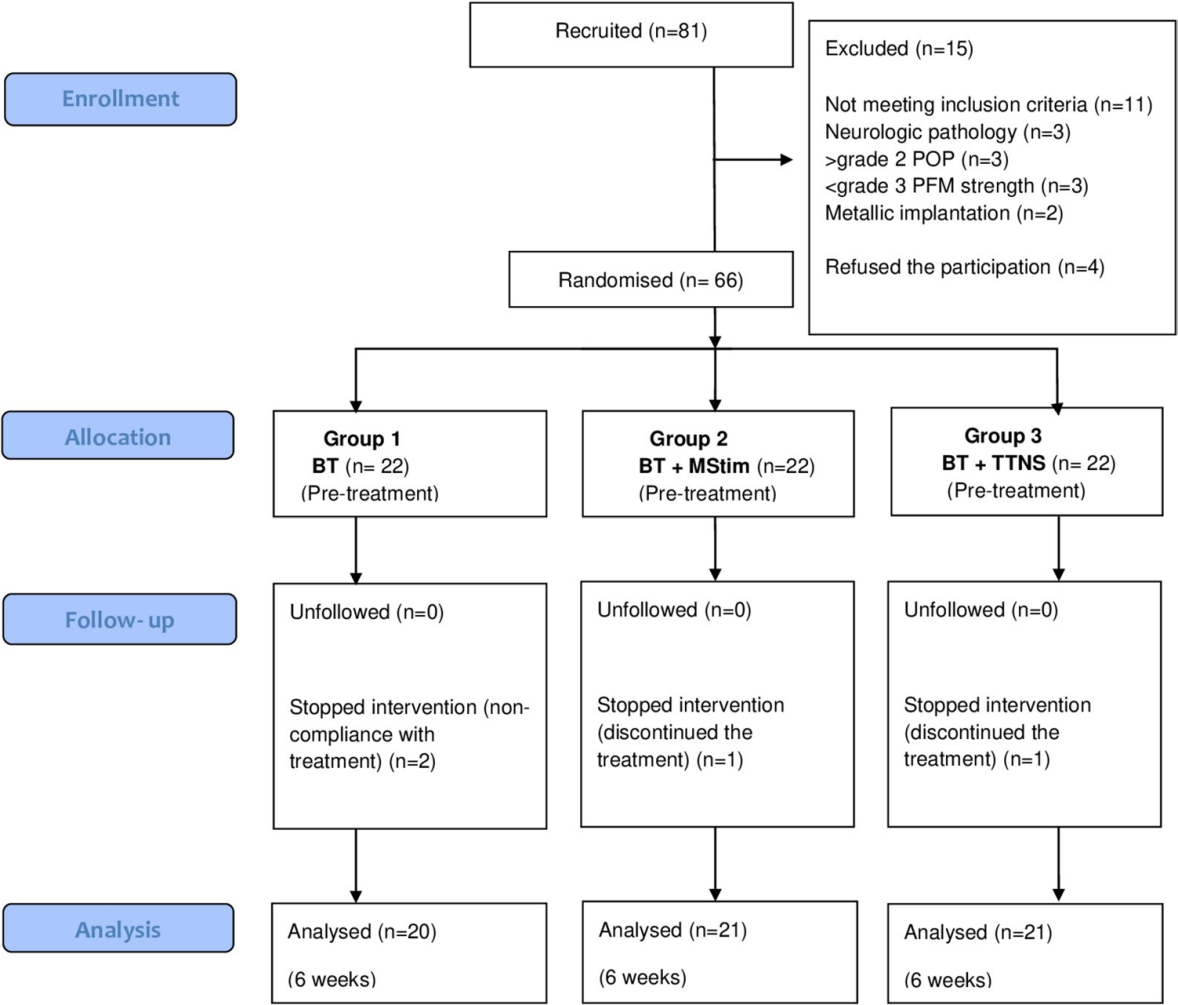
CONSORT participant flow diagram for randomized, controlled trials of nonpharmacologic treatment. *BT*, Bladder training; *PFM*, pelvic floor muscle; *POP*, pelvic organ prolapse; *MStim*, Magnetic stimulation; *TTNS*, Transcutaneous tibial nerve stimulation

Eighty-one women were assessed for eligibility. After initial evaluation, 66 women met the inclusion and exclusion criteria. The CONSORT flow chart of participant recruitment is shown in Fig. 3. Following randomization, each of the three groups consisted of 22 patients. Two women in group 1 withdrew from the study due to noncompliance with the BT program. One woman in group 2 withdrew for personal social reasons, and one woman in group 3 withdrew due to transportation issues, which prevented her from attending the treatment. Data from these participants were excluded from the analysis (Fig. 3).

The three groups were comparable in terms of demographic and clinical characteristics. There were no statistically significant differences among the groups regarding age, BMI, duration of incontinence, education level, smoking status, and menopausal status. Additionally, the consumption of tea, coffee, and alcohol, as well as obstetric history (parity, delivery type, and episiotomy), were similar across the groups (*p* > 0.05) (Table 2). Furthermore, the groups were comparable at baseline with respect to incontinence severity, voiding frequency, incontinence episodes, nocturia, number of pads used, symptom severity, and QoL (*p* > 0.05) (Table 3).

**Table 2:** Sample characterization

	Group 1 *n* = 20	Group 2 *n* = 21	Group 3 *n* = 21	*P*^1^	*P*^2^
Age (year) (mean ± SD)	60.25 (± 9.35)	55.90 (± 8.64)	59.33 (± 11.04)	0.294	
Height (cm) (mean ± SD)	158.25 (± 5.40)	158.19 (± 5.49)	158.66 (± 7.18)	0.938	
Weight (kg) (mean ± SD)	76.55 (± 13.16)	75.71 (± 14.91)	76.90 (± 13.63)	0.804	
BMI (kg/m^2^) (mean ± SD)	30.45 (± 4.92)	30.31 (± 6.09)	30.61 (± 5.34)	0.935	
Duration of incontinence (month) (mean ± SD)	96.10 (± 72.49)	113. 80 (± 73.52)	88.33 (± 89.26)	0.182	
Education, *n* (%)					
Primary	11 (55)	15 (71.5)	15 (71.5)		
High	4 (20)	2 (9.5)	4 (19)		
> High	5 (25)	4 (19)	2 (9.5)		0.636
Smoking, *n* (%)					
N	19 (95)	17 (81)	19 (90.5)		
Yes	1 (5)	4 (19)	2 (9.5)		0.606
Tea/day, *n* (%)					
No	1 (5)	1 (4.8)	1(4.8)		
1–2 cup	10 (50)	6 (28.6))	2 (9.5)		
≥ 3 cup	9 (45)	14 (66.7)	18 (85.7)		0.079
Coffee/day, *n* (%)					
No	9 (45)	3 (14.3)	9 (42.9)		
1–2 cup	10 (50)	15 (71.4)	11 (52.4)		
≥ 3 cup	1 (5)	3 (14.3)	1 (4.8)		0.190
Alcohol, *n* (%)					
No	19 (95)	19 (90.5)	20 (95.2)		
Yes	1 (5)	2 (9.5)	1 (4.8)		0.780
Delivery, *n* (%)					
Yok	1 (5)	1 (4.8)	-		
1–3	16 (80)	16 (76.2)	19 (90.5)		
≥ 4	3 (15)	4 (19)	2 (9.5)		0.742
Delivery type, *n* (%)					
NSVD	17 (85)	19 (90.5)	16 (76.2)		
Sectio	2 (10)	1 (4.8)	5 (23.8)		0.351
Episiotomy, *n* (%)					
Hayır	9 (45)	10 (47.6)	7 (33.3)		
Evet	11 (55)	11 (52.4)	14 (66.7)		0.608
Menopausal status, *n* (%)					
Premenopause	4 (20)	8 (38.1)	3 (14.3)		
Postmenopause	16 (80)	13 (61.9)	18 (85.7)		0.171
HRT, *n* (%)					
No	17 (85)	19 (90.5)	16 (76.2)		
Yes	3 (15)	2 (9.5)	5 (23.8)		0.447

Group1, bladder training; Group2, bladder training plus magnetic stimulation (MStim); Group3, bladder training plus transcutaneous tibial nerve stimulation (TTNS); *HRT*, hormon replacement treatment; *BMI*, body mass index; *NSVD*, normal spontaneous vaginal delivery; *SD*, standart deviation; P^1^, Kruskal–Wallis test; P^2^, Pearson χ2 test

**Table 3:** The comparison between groups of patient’s evaluation parameters (mean ± standart deviation)

	Group 1 *n* = 20	Group 2 *n* = 21	Group 3 *n* = 21	*P*	Mann–Whitney-U test with Bonferroni correction
Incontinence severity—24 h pad test (gr)					
Pre-treatment	55.5 (77.5–14.5)	42.0 (58.5–19.25)	50.0 (104.0–14.5)	0.571	
Post-treatment (6th week)	34.0 (67.25–5.0)^a^	9.0 (22.0–1.25)^a^	8.0 (22.5–0.5)^a^	0.015	Group1 < Group2^b^ Group1 < Group3^b^
Bladder diary					
Voiding frequency (times/day)					
Pre-treatment	11.0 (12.0–10.25)	12.0 (13.0–11.0)	11.0 (12.0–10.0)	0.104	
Post-treatment (6th week)	8.0 (10.0–7.0)^a^	8.0 (10.0–7.0)^a^	6.0 (7.0–5.0)^a^	0.000	Group1 < Group3^b^ Group2 < Group3^b^
Nocturia (times/night)					
Pre-treatment	2.5 (3.0–2.0)	3.0 (3.0–2.0)	3.0 (3.0–2.5)	0.409	
Post-treatment (6th week)	1.0 (2.0–1.0)^a^	1.0 (1.5–0.5)^a^	1.0 (2.0–1.0)^a^	0.202	
Incontinence episodes (times/day)					
Pre-treatment	4.5 (5.75–3.0)	4.0 (5.0–2.0)	4.0 (5.0–2.5)	0.455	
Post-treatment (6th week)	3.0 (5.75–1.25)^a^	1.0 (2.0–0.5)^a^	1.0 (2.0–0.0)^a^	0.002	Group1 < Group2^b^ Group1 < Group3 ^b^
Number of pads (per day)					
Pre-treatment	3.0 (3.0–1.25)	2.0 (3.0–1.0)	2.0 (4.0–1.0)	0.646	
Post-treatment (6th week)	2.0 (3.0–1.0)	1.0 (1.0–1.0)^a^	1.0 (2.0–0.5)^a^	0.003	Group1 < Group2^b^ Group1 < Group3^b^
Symptom severity OAB-V8					
Pre-treatment	34.0 (37.0–29.0)	31.0 (37.0–26.0)	30.0 (35.0–25.0)	0.513	
Post-treatment (6th week)	26.0 (31.5–23.0)^a^	16.0 (20.5–8.5)^a^	17.0 (21.0–10.0)^a^	0.000	Group1 < Group2^b^ Group1 < Group3^b^
Quality of life-IIQ7					
Pre-treatment	19.0 (20.0–17.25)	17.0 (19.0–12.5)	18.0 (19.5–13.0)	0.153	
Post-treatment (6th week)	16.0 (18.0–14.0)^a^	11.0 (13.5–3.0)^a^	9.0 (12.0–4.0)^a^	0.000	Group1 < Group2^b^ Group1 < Group3^b^
Treatment satisfaction (1–5)					
Post-treatment (6th week)	4.0 (4.0–3.0)	5.0 (5.0–4.0)	4.0 (5.0–4.0)	0.000	Group1 < Group2^b^ Group1 < Group3^b^

Values are presented as median (interquartile range): *IQR* 3rd quartile–1st quartile; Group1, bladder training; Group2, bladder training plus magnetic stimulation (MStim); Group3, bladder training plus transcutaneous tibial nerve stimulation (TTNS); *OAB-V8*, Overactive Bladder Questionnaire; *IIQ-7*, Incontinence impact questionnaire; *P*, Kruskall-Wallis test; ^a^, *p* < 0.05: compare with baseline values; ^b^, *p* < 0.0167: Mann–Whitney-U test with Bonferroni correction

At the end of the treatment period, all groups demonstrated statistically significant improvements from baseline in all measured parameters, except for the number of pads used in group 1 (*p* < 0.05) (Table 3). Both stimulation groups (groups 2 and 3) showed significantly greater improvements in incontinence severity, incontinence episodes, number of pads used, symptom severity, and QoL compared to the control group (group 1) (*p* < 0.0167). No significant differences were observed between group 2 and group 3 for these parameters (Table 3). Voiding frequency improved significantly in group 3 compared to both group 1 and group 2 (*p* < 0.0167). No significant difference in voiding frequency was found between group 1 and group 2 (Table 3). There were no significant differences between the three groups in terms of nocturia (*p* > 0.05). Treatment satisfaction scores were significantly higher in both stimulation groups compared to the control group (Table 3).

Positive response rate at week 6, the primary outcome measure, was greater in groups 2 and 3 than group 1 (14 (66.7%) and 19 (90.5%) vs. 7 (35.0%), *p* = 0.001). Statistically significant high values were found in positive response rate in groups 2 and 3 compared to group 1 at week 6 (odds ratio 0.269 (95%CI 0.074–0.979), *p* = 0.013, and odds ratio 0.057 (95%CI 0.010- 0.317), *p* < 0.001, respectively). In comparing groups 2 and 3, positive response was similar at week 6 (odds ratio 0.210 (95%CI 0.038–1.171), *p* = 0.130).

MStim and TTNS were well-tolerated. No serious adverse events were reported. In group 2, two women (9.5%) experienced temporary discomfort related to pelvic floor pain, and one woman (4.7%) reported malaise. In group 3, two women (10%) experienced temporary ecchymosis at the electrode application site.

## Discussion

In this prospective, randomized controlled trial, we compared the efficacy of TTNS and MStim on QoL and clinical factors associated with incontinence in women with iOAB. Our findings demonstrate significant improvements in incontinence severity, voiding frequency, incontinence episodes, nocturia, symptom severity, and QoL at the 6-week assessment in all groups compared to baseline. Notably, both stimulation groups (BT plus MStim and BT plus TTNS) exhibited significantly greater improvements in incontinence severity, incontinence episodes, number of pads used, symptom severity, and QoL, along with higher treatment satisfaction and better positive response rates compared to the BT-only group. Furthermore, BT plus TTNS was significantly more effective in reducing voiding frequency than BT plus MStim.

Our observed positive response rate of 35% in the BT-only group aligns with previous research demonstrating improvement rates ranging from 35–63% in BT interventions for iOAB [4, 5, 8, 24, 31]. This variation likely stems from differences in BT protocols and outcome measures across studies. Similarly, our positive response rates for the MStim plus BT and TTNS plus BT groups (66.7% and 90.5%, respectively) are consistent with prior findings in women with iOAB [7, 8, 18].

Our study supports previous research indicating the superiority of both TTNS plus BT and MStim plus BT over BT alone in improving QoL and incontinence-related clinical parameters in women with iOAB [7, 8, 10]. Consistent with a previous study [18], we found that adding MStim to BT did not provide additional benefit in reducing voiding frequency.

While both TTNS combined with BT and MStim combined with BT were more effective than BT alone, the lack of a statistically significant difference between TTNS and MStim—except for voiding frequency—should not be interpreted as clinical equivalence. These two neuromodulation techniques differ substantially in terms of their application methods. Despite comparable clinical efficacy and high levels of treatment satisfaction, appropriate patient selection remains essential. Individual needs and contraindications should guide therapy choice to optimize outcomes and enhance adherence. Through careful patient selection, these noninvasive approaches can be effectively utilized to minimize side effects, reduce complications, and improve treatment success. Further high-quality studies are warranted to better define the respective roles of TTNS and MStim in managing iOAB in women, thereby aiding clinicians in making evidence-based treatment decisions. Although our study did not include a cost analysis of the equipment and devices used in both interventions, future studies should address treatment costs and cost-effectiveness. On the basis of current observations, TTNS may offer economic advantages; however, this requires formal validation. Importantly, long-term treatment goals—including the durability of therapeutic effects, sustainability, and maintenance protocols—should be key areas of focus in future research.

This study has several notable strengths. It is the first prospective randomized controlled trial to directly compare the effectiveness of MStim and TTNS in women with iOAB. The results clearly demonstrate that both neuromodulation techniques, when used in combination with bladder training, are more effective than bladder training alone. Moreover, the comparable efficacy between MStim and TTNS is highlighted, with TTNS showing a greater reduction in voiding frequency. These findings contribute meaningful evidence to the existing literature and may assist clinicians and patients in selecting the most appropriate treatment options for iOAB.

The limitations of our study include the relatively small sample size and single-center design, which may affect the generalizability of the findings. There is no long-term follow-up, which limits our understanding of the durability of treatment effects. The absence of a cost-effectiveness analysis restricts the ability to evaluate the economic impact of the interventions. The lack of patient blinding may have introduced bias, and increased face-to-face interaction with healthcare professionals in the stimulation groups could have influenced the outcomes. In addition, excluding women with poor PFM strength limits the applicability of the results to a wider population. Although the stimulation parameters for both TTNS and MStim were consistent with previous literature, they may not be optimal, and further studies are needed to compare different stimulation frequencies (e.g., daily, twice weekly, weekly) and durations. Additionally, the maximum tolerable stimulation intensity and stimulation current intensity were not systematically recorded for all participants and sessions. As a result, the variability in stimulation intensity could not be assessed, which may have influenced treatment outcomes. Future studies should include detailed records of stimulation intensity to better understand its impact on clinical efficacy and treatment variability. As fluid intake habits were not controlled or normalized in this study, the potential impact of fluid consumption on voiding frequency was not considered. This limitation may affect the generalizability of the results, and future studies should consider controlling for fluid intake to mitigate its influence on urinary outcomes. Despite these limitations, our study provides valuable insights into the management of iOAB in women and offers a basis for future research.

## Conclusion

Both MStim and TTNS, when added to BT, are more effective than BT alone in managing iOAB. While TTNS demonstrated superiority over MStim in reducing voiding frequency, both treatments showed similar efficacy in improving other key outcomes. These include incontinence severity, number of incontinence episodes and pads used, symptom severity, QoL, treatment satisfaction, and positive response rates.

## Data Availability

Data is not open to public but can be obtained from the corresponding author upon reasonable request.

## Abbreviations

BT: Bladder training
iOAB: Idiopathic overactive bladder
MStim: Magnetic stimulation
QoL: Quality of life
PFM: Pelvic floor muscle
PTNS: Percutaneous tibial nerve stimulation
TTNS: Transcutaneous tibial nerve stimulation
